# Kinetic, Thermodynamic, Physicochemical, and Economical Characterization of Pectin from *Mangifera indica* L. cv. Haden Residues

**DOI:** 10.3390/foods10092093

**Published:** 2021-09-04

**Authors:** Sergio Valdivia-Rivera, Iván Emanuel Herrera-Pool, Teresa Ayora-Talavera, Manuel Alejandro Lizardi-Jiménez, Ulises García-Cruz, Juan Carlos Cuevas-Bernardino, José Manuel Cervantes-Uc, Neith Pacheco

**Affiliations:** 1 Centro de Investigacion y Asistencia en Tecnologia y Diseño del Estado de Jalisco, Sede Sureste, Parque Cientifico Tecnologico de Yucatan, Km 5.5, Carretera Sierra Papacal-Chuburna Puerto, Merida 97302, Yucatan, Mexico; sevaldivia_al@ciatej.edu.mx (S.V.-R.); ivherrera_al@ciatej.edu.mx (I.E.H.-P.); tayora@ciatej.mx (T.A.-T.); 2 CONACYT, Universidad Autonoma de San Luis Potosi, Sierra Leona 550, Lomas Segunda Seccion, San Luis Potosi 78210, San Luis Potosi, Mexico; manuel.lizardi@uaslp.mx; 3 Centro de Investigacion y de Estudios Avanzados-Merida, Antigua Carretera a Progreso Km 6, Cordemex, Loma Bonita Xcumpich, Mérida 97310, Yucatan, Mexico; norbertoulisesg@gmail.com; 4 CONACYT, Centro de Investigacion y Asistencia en Tecnologia y Diseño del Estado de Jalisco, Sede Sureste, Parque Cientifico Tecnologico de Yucatan, Km 5.5, Carretera Sierra Papacal-Chuburna Puerto, Merida 97302, Yucatan, Mexico; jcuevas@ciatej.mx; 5 Centro de Investigacion Cientifica de Yucatan, Unidad de Materiales, Calle 43 No. 130 x 32 y 34, Chuburna de Hidalgo, Merida 97205, Yucatan, Mexico; manceruc@cicy.mx

**Keywords:** Haden mango (*Mangifera indica* L.), pectin, thermodynamic, extraction kinetic, physicochemical characterization, citric acid extraction

## Abstract

The effect of temperature (60, 70, 80, and 90 °C) and time (30, 45, 60, 75, and 90 min) on citric acid extraction of Haden mango (*Mangifera indica* L. cv. Haden) peel pectin was evaluated in the present study. In order to obtain a better understanding of both the extraction process and the characteristics of the pectin (obtained from an agro-industrial waste) for a future scaling process, the following characterizations were performed: (1) Kinetic, with the maximum extraction times and yields at all evaluated temperatures; (2) thermodynamic, obtaining activation energies, enthalpies, entropies, and Gibbs free energies for each stage of the process; (3) physicochemical (chemical analysis, monosaccharide composition, degree of esterification, galacturonic acid content, free acidity, Fourier-transform infrared spectroscopy, thermogravimetric and derivative thermogravimetric analyses); and (4) economical, of the pectin with the highest yield. The Haden mango peel pectin was found to be characterized by a high-esterified degree (81.81 ± 0.00%), regular galacturonic acid content (71.57 ± 1.26%), low protein (0.83 ± 0.05%) and high ash (3.53 ± 0.02%) content, low mean viscometric molecular weight (55.91 kDa), and high equivalent weight (3657.55 ± 8.41), which makes it potentially useful for food applications.

## 1. Introduction

Mexico is one of the main worldwide producers of Mango (*Mangifera indica* L.) with an annual mean production of 1654 t [1,2]. The two main industrial residues derived from mango are the peel and kernel, which represent 30 to 55% of the total fresh mango weight and generally are discarded into the environment, creating health, economic, and environmental issues [3,4]. As the mango peel and seed are composed of various polysaccharides, lipids, phytochemicals, and enzymes of industrial relevance, such as pectin [3,5], efforts to take advantage of its by-products and waste have increased.

Pectin is a polysaccharide consisting mainly of galacturonic acid (GalA) in which three regions are distinguished: homogalacturonan (HG), where GalA units are covalently linked in (1-4)-α-D bonds; rhamnogalacturonan I (RGI), consisting in GalA linked in (1-4)-α-D bonds with L-rhamnose residues interspersed with (1-2)-α-L bonds; and rhamnogalacturonan II (RGII), formed by HG linked in side chains to GalA units [6]. The HG region can be partially esterified at the C-6 carboxyl and acetylated at *O*-2 or *O*-3. The frequency or quantity of methyl groups gives different degrees of esterification (DE) to pectin, which determines its techno-functional properties [7,8]. When DE of the pectin is >50%, it is considered as high methoxylation, while DE < 50% indicates a low methoxylation polysaccharide [7,9]. The RGI and RGII regions of pectin may contain neutral sugar residues, such as arabinose, galactose, rhamnose, xylose, or glucose, although RGII usually has a highly homogeneous structure compared to RGI [10]. This polysaccharide usually has an average molecular weight (AMW) of between 50 and 150 kDa [11]. Pectin is widely used in the food and pharmaceutical industries due to its versatility, as it can be used for the formation of gels, as a thickener, to provide physical stability, as an excipient, or as an encapsulating agent to control the release of active ingredients [6,10,12], although pectin has also been used for environmental purposes, such as an adsorbent to remove metal ions from water [13]. For most of these industrial applications, especially in food industry, a high DE pectin is preferred as it will provide greater flexibility (e.g., for film elaboration) and facilitate manipulation [10,14] due to its molecular conformation with a lower presence of “hairy” regions (RGI and RGII) [15]. Another important factor to consider is that the intrinsic viscosity [η] and DE will influence pectin’s ability to form gels. In addition, it is possible to obtain a pectin with a greater gelling capacity and strong hydrophobic forces if it has high DE [14]. 

Traditionally, pectin has been industrially extracted from three sources: apple pulp (14.00%), citrus peel (85.50%), and beet pulp (0.50%). Despite this, there are other sources from which pectin can be obtained with physicochemical properties that depend on its origin, such as the degree of esterification, galacturonic acid content, neutral sugar composition, and molecular weight [16,17]. Extraction percentages ranging from 4.20% (apple variety Granny Smith) to 26.30% (lime peel) are achieved from traditional sources of pectin with DE of 52.51% for apple, and 82.20% for lime peel. However, there are other sources, such as tomato peel (32.60%), watermelon rind (19.00 to 21.00%), or mango peel (17.15%), where DE of 85.43–88.38% have been reported that would be worth exploring because pectin yield may vary depending on the pectin source [17], extraction method, and temperature used [18]. Usually, in the extraction of pectin, mineral acids that generate corrosive effluents are used (e.g., sulfuric, hydrochloric, and nitric acid), so in order to avoid environmental contamination, work has begun with other types of acids, such as citric acid, which is friendlier to the environment [12,19]. Additionally, citric acid extraction has been reported to produce a higher amount of methoxy groups, and improve the viscosity and molecular weight compared to other pectins from the same source but extracted with conventional methods that can be used in other important industry areas [18,20]. 

Citric acid extraction has become more common in recent years for pectin obtention, due to the advantages reported. Pectin has been extracted from hawthorn wine pomace waste [21], apple pomace [22], or even from mango varieties like Tainong [18] and Keitt [23] using citric acid. Some studies have even reported optimization of the citric acid extraction process coupled with ultrasound-assisted extraction to recover pectin from fruits like passion fruit [19]. However, few studies have reported kinetic and thermodynamic characterization of pectin extraction in acid medium [14,24] and even less have used citric acid [25], which is relevant for evaluation, optimization, and scale-up of the process [26]. To the best of our knowledge, no kinetic and thermodynamic parameters of Haden mango pectin citric acid extraction have been previously reported.

The objective of the present work was to obtain pectin from *Mangifera indica* L. cv. Haden grown in Tapachula, Chiapas for mango residue valorization, and to characterize the kinetic and thermodynamic process of its extraction with citric acid, as well as to identify the chemical and physicochemical characteristics of the pectin obtained for its better understanding and study. The novelty of this works lies in the extraction and characterization of pectin from residues of an understudied mango variety, extracted with a non-conventional solvent, such as citric acid, in order to obtain useful kinetic and thermodynamic parameters for process efficiency analysis and its future scalability.

## 2. Materials and Methods

### 2.1. Materials

The Haden mango (*Mangifera indica* L. cv. Haden) samples were harvested in Canton de los Llanos (14°46′04.1″ N, 92°20′40.8″ W) in Tapachula, Chiapas, Mexico on 27 April of 2019. The samples were transported to Merida, Yucatan, Mexico on 3 May of 2019 and stored in a dark room at 4 °C until 6 May. The samples had a stage of maturity 4 at time of processing [27]. The citric acid used was food grade (Weifang Ensign Industry Co., Shandong, China). Ethanol (96%), potassium sulfamate, sodium tetraborate, sulfuric acid, 3-phenylphenol, NaOH, D-Galacturonic acid, hydrochloric acid, phenolphthalein, and NaCl were purchased from Sigma Aldrich (Darmstadt, Germany).

### 2.2. Obtaining Haden Mango Peel Flour

The mango fruits were washed with a commercial dish detergent and the peel was removed manually. The peels were dried in a tray dehydrator (Jersa, Mexico city, Mexico) at 60 °C until a constant weight was obtained (8 h). The dried peels were crushed in a hammer mill (Pulvex, Mexico city, Mexico) and sieved to a particle size of 0.5 mm. The mango flour obtained was stored away from light and moisture at −4 °C.

### 2.3. Pectin Extraction 

**Figure 1:**
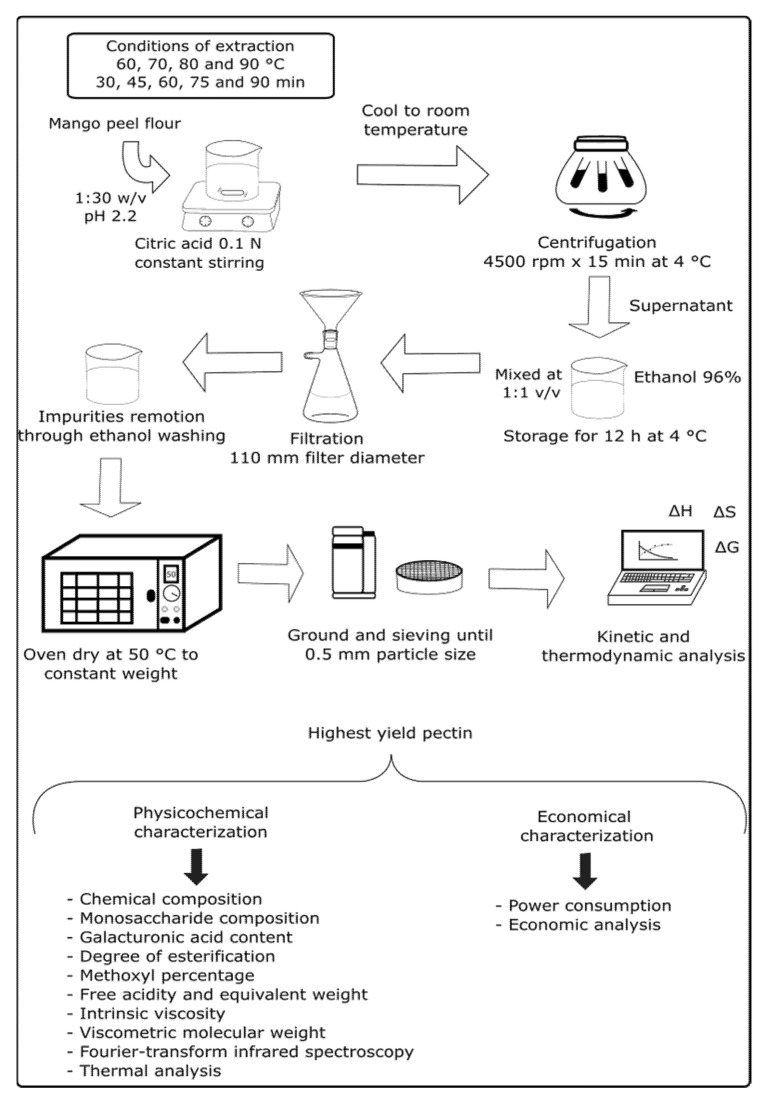
Extraction strategy of Haden mango peel pectin and further analyses of the highest yield pectin.

For the extraction of pectin, a previous reported methodology [12] was used with modifications: an acid hydrolysis was used with citric acid (0.1 N), in a ratio of 1:30 *w*/*v* using 1 g of mango flour per extraction. The citric acid solution was preheated to a tested temperature, and the mango flour was added and allowed to react for a defined tested time with constant stirring. The pH after mango flour addition was 2.2. The mixture was allowed to cool and centrifuged in a SL 40R centrifuge (Thermo Scientific, Waltham, MA, USA) at 4500 rpm for 15 min at 4 °C. The supernatant was mixed with 96% ethanol (1:1 *v*/*v*) and kept at 4 °C for 12 h. The pectin samples were filtered with a Whatman filter (GE Healthcare System, Chicago, IL, USA) with a 110 mm diameter, washed with ethanol repeatedly, and dried in an OV-12 vacuum oven (Jeio Tech, Seoul, Korea) at 50 °C until a constant weight was reached. Pectin samples were ground with a GX4100 coffee mill (KRUPS, Solingen, Germany and sieved to a 0.5 mm particle size with a 0.5 mm stainless steel mesh. For this work, during the extraction of the pectin, 4 heating temperatures (60, 70, 80, and 90 °C) and 5 reaction times (30, 45, 60, 75, and 90 min) were tested. Figure 1 shows a schematic graphic of the full extraction strategy and further analyses of the Haden mango peel pectin. 

The extraction yield was determined as follows: (dry weight of the pectin × 100)/dry weight of the mango flour used.

#### Statistical Analysis

The significance of differences between the means of the pectin yield extraction was evaluated by analysis of variance (ANOVA) and the Tukey’s test at *p* < 0.05, using the statistical software Jamovi (Version 1.1.9.0). Each pectin extraction was performed in triplicate. 

### 2.4. Kinetic Characterization 

A kinetic model of pectin extraction consisting of two simultaneous reactions was used: Diffusion of pectin, or protopectin, from mango peel flour (mediated by reaction rate constant *k*_1_) and partial degradation of dissolved pectin (mediated by reaction rate constant *k*_2_) in the acid media [25,26], i.e.,:

$$\mathrm{Mango}\;\mathrm{peel}\;\mathrm{pectin}\;\overset{k_{1}}{\Rightarrow }\;\mathrm{Dissolved}\;\mathrm{pectin}\;\overset{k_{2}}{\Rightarrow }\;\mathrm{Degraded}\;\mathrm{pectin}$$

In this model, the pectin or protopectin inside the mango peel that can be extracted under certain conditions at time *t* is called *z*(*t*). The amount of dissolved pectin in the citric acid solution by the effect of the extraction is *y*(*t*). The pectin possibly degraded by the effect of time, temperature, acid, or all conditions used in the extraction is represented as *q*(*t*). The most basic model with which it can be represented is with a first-order reaction, like Equations (1)–(3):

(1) $$dz\left(t\right)/dt=-k_{1}z\left(t\right)\;$$

(2) $$dq\left(t\right)/dt=k_{2}y\left(t\right)$$

(3) $$dy\left(t\right)/dt=k_{1}z\left(t\right)-k_{2}y\left(t\right)$$

Furthermore, the percentage of pectin that can be extracted from the mango peel under certain conditions is referred to as *P_E_*. Therefore, it is possible to calculate it using Equation (4):

(4) $$P_{E}=z\left(t\right)+q\left(t\right)+y\left(t\right)$$

The yield of extraction or extracted pectin *p*(*t*) can be described by Equation (5):

(5) $$p\left(t\right)=P_{E}k_{1}/\left(k_{2}-k_{1}\right)\left(\exp\left(-k_{1}t\right)-\exp\left(-k_{2}t\right)\right)$$

The dissolved pectin *y*(*t*) can be obtained by Equation (6):

(6) $$y\left(t\right)=p\left(t\right)+q\left(t\right)=P_{E}\left(1-\exp\left(-k_{1}t\right)\right)$$

From the above equations, the maximum value of the pectin yield (*y_max_*) can be obtained with Equation (7), and the time (*t_max_*) in which *y_max_* is reached using Equation (8):

(7) $$y_{max}=P_{E}{\left(k_{2}/k_{1}\right)}^{\left(k_{2}/k_{1})/(1-k_{2}/k_{1}\right)}\;(7)$$

(8) $$t_{max}=ln(k_{1}/k_{2})/\left(k_{1}-k_{2}\right)$$

### 2.5. Thermodynamic Characterization

Equation (5) was solved by non-linear regression using the trust region algorithm in Matlab software (R2017b, student version); the constants *k*_1_ and *k*_2_ were obtained. Using the values of constants *k*_1_, *k*_2_ and the Arrhenius equation, the activation energies (*Ea*) at different temperatures were obtained. Activation energy was calculated using the values of *lnk* vs. 1/*T* graph, where the slope is equal to −*Ea*/*R* [28]. In the Arrhenius Equation (9), *R* is the gas constant with a value of 8.324 J/mol. *K* and *T* are given in Kelvin:

(9) lnk=lnA−Ea/RT

To calculate the thermodynamic activation parameters of enthalpy (Δ*H*^#^), entropy (Δ*S*^#^), and Gibbs free energy (Δ*G*^#^), the Eyring Equation (10) was used:

(10) $$ln\left(k/T\right)=ln\left(kb/h\right)+\Delta S^{\#}/R-\Delta H^{\#}/RT$$

To obtain Δ*H*^#^ and Δ*S^#^,* a *lnk*/*T* vs. 1/*T* graph was plotted, where the slope is equal to Δ*H*^#^/*R* and the intercept with the origin is Δ*S*^#^/*R* (Equation (11)). Furthermore, obtaining the equilibrium constant *F*, which is equal to *Y_max_*/*z*(*t*), it is possible to obtain the values of enthalpy (Δ*H*), entropy (Δ*S*), and Gibbs free energy (Δ*G*):

(11) lnF=−ΔH/RT+ΔS/R

Plotting *lnF* vs. 1/*T*, the slope is equal to Δ*H*/*R* and the intercept with the origin is Δ*S*/*R*. 

The average of the yields obtained for each experiment treatment from Section 2.3 were used as inputs for the kinetic and thermodynamic calculations. 

### 2.6. Physicochemical Characterization

#### 2.6.1. Chemical Composition

The moisture and ash contents were obtained by gravimetry using porcelain crucibles at constant weight, according to the methods of the 18th edition of the AOAC: 934.01 for moisture, and 942.05 for ash [29]. The crude protein content was obtained on a DKL Fully Automatic Digestion Units and UDK129 Distillation Unit (Velp Scientifica, Usmate Velate, Italy) using Module 9 and the manufacturer’s instructions and a N × 6.25 factor to determine the protein content. 

#### 2.6.2. Monosaccharide Composition 

The monosaccharide composition was determined for qualitative analysis by ultra performance liquid chromatography (UPLC) using hydrolysis and 1-phenyl-3-methyl-5-pyrazolone (PMP) derivatization methodology as described elsewhere [30,31,32], with brief modifications:

In total, 3 mg of dry pectin were hydrolyzed in 200 µL of trifluoroacetic acid (2 M) for 3 h at 85 °C. At the end of hydrolysis, the sample was neutralized with NaOH (0.1 M) and dried. The hydrolyzed was re-suspended in 10 µL of PMP (0.5 M in methanol) and 10 µL of NaOH (0.3 M) and incubated at 70 °C for 30 min for derivatization. After derivatization, the samples were neutralized with HCl (0.3 M) and dried. The sample was re-suspended in 1.5 mL of water and PMP residues were extracted three times with 6 mL of chloroform. The sample was filtered with a nylon 0.2 µm Millex-GM Millipore filter (Merck, Darmstadt, Germany) prior to UPLC analysis. Monosaccharide standards were derivatized following the same methodology.

For the UPLC analysis, an ACQUITY UPLC equipment (Waters, Wilmslow, England) with UV detection was used. Two mobile phases were used: A (15% acetonitrile, 85% potassium phosphate buffer 0.05 M) and B (40% acetonitrile, 60% potassium phosphate buffer 0.05 M). The temperature of the column was 27 °C, the flow rate was constant at 0.2 mL/min, and four gradients for the mobile phases were used (Table 1).

The total analysis time was 30 min and 2 µL of sample were injected. The UV detector worked in the λ range of 210 to 400 nm in 2 channels: 254 nm and 242 nm with 4.8 nm of resolution. 

Additionally, hydrolyzed pectin samples were suspended in 1 mL of distilled water and used for the determination of the glucose + fructose, and fructose content by reflectometry using the Reflectoquant^®^ total sugar test (glucose and fructose) and Reflectoquant^®^ glucose test kits (Merck, Darmstadt, Germany). The manufacturer’s instructions were followed. 

#### 2.6.3. Galacturonic Acid Content 

The galacturonic acid content was determined in a spectrophotometer Biomate 3S (Thermo Scientific, Waltham, MA, USA), using a previous calibration curve with D-galacturonic acid as the standard (2.5 to 125 µg/mL) at 525 nm. The procedure was as follows: it started with a volume of 0.4 mL of pectin solution at a concentration of 100 µg mL^−1^ and 40 µL of potassium sulfamate (4 M at pH 1.6) were added. Then, 2.5 mL of sodium tetraborate (75 mM) in sulfuric acid were added and the resulting mixture was vortex homogenized for 1 min. The hot sample was cooled in an ice bath. Subsequently, it was placed in a water bath for 15 min and the ice bath cooling was repeated for 1.5 min. Finally, 80 µL of 3-phenylphenol 0.15% (*w*/*v*) in NaOH 0.5% were added and it was mixed in vortex for 3 min. The sample was analyzed in the spectrophotometer [12,33]. For the analysis and calculation, the Food and Agricultural Organization (FAO) and the European Union (EU) were considered to establish the galacturonic acid content calculated based on the ash and moisture-free mass [34].

#### 2.6.4. Degree of Esterification 

For the determination of the degree of esterification (%*DE*), a previous reported methodology was used [35] with minor modifications: 500 mg of pectin were moistened with 2 mL of ethanol and dissolved in 100 mL of distilled carbon dioxide-free water (by heating). After dissolution of the sample, 5 drops of phenolphthalein were added and the sample was titrated with 0.5 M NaOH, neutralizing the free carboxylic acids of galacturonic acid. The spent volume was reported as the initial volume (*V*_1_). Subsequently, 10 mL of 0.5 M NaOH were added, the sample was vigorously stirred for 15 min for hydrolysis, and 10 mL of 0.5 M hydrochloric acid were added under vigorous stirring until the pink color of the solution disappeared. Finally, the sample was titrated with 0.5 M NaOH and vigorously stirred until the sample turned pink (*V*_2_). The degree of esterification of pectin was calculated using Equation (12):

(12) $$\%DE=[V_{2}/\left(V_{1}+V_{2}\right)]\times 100$$

#### 2.6.5. Methoxyl Percentage

The methoxyl percentage (%*MeO*) was determined considering that the methoxyl content in 100% esterified pectin is 16.32% [12,33], so %*MeO* was obtained with Equation (13):

(13) $$\%MeO=\frac{16.32}{100}\times \%DE$$

#### 2.6.6. Free Acidity and Equivalent Weight

The free acidity was determined according to a previous reported methodology [36]: 0.1 g of dry pectin were weighed and dissolved in 20 mL of distilled water at 40 °C with constant stirring. The resulting solution was titrated with a 0.1 M NaOH solution and 2 drops of 1% phenolphthalein in ethanol. Free acidity was calculated with Equation (14):

(14) $$\mathrm{Free}\;\mathrm{acidity}=\frac{\mathrm{Spend}\;\mathrm{volume}\;\mathrm{of}\;\mathrm{NaOH}\;\left(\mathrm{mL}\right)\times \mathrm{NaOH}\;\mathrm{concentration}\left(M\right)\;}{\mathrm{Sample}\;\mathrm{weight}\;\left(g\right)}$$

The results were expressed in terms of milliequivalents of free carboxyl/gram. To determine the equivalent weight, Equation (15) was used:

(15) $$\mathrm{Eq}.\;\mathrm{weight}=\frac{\mathrm{Sample}\;\mathrm{weight}\;\left(g\right)\times 1000}{\mathrm{NaOH}\;\mathrm{mEq}}$$

The NaOH mEqs were calculated considering the normality used and the spent volume in mL of NaOH.

#### 2.6.7. Intrinsic Viscosity and Viscometric Molecular Weight 

The intrinsic viscosity [η] was determined according to Trujillo-Ramirez et al. (2018): Standard solutions (0.1% *w*/*v*) were prepared with pectin powder in deionized water (100 mM NaCl) under magnetic stirring for 30 min until its complete dissolution at room temperature. Subsequently, the samples were stored at 25 °C for 24 h to dehydrate. Each standard solution was diluted in a concentration range of 0.001–0.1 g 100 mL^−1^. Samples were vortexed and allowed to stand for 15 min prior to viscosity measurement. Viscosity was determined with a Discovery HR-2 rheometer (TA Instruments, New Castle, DE, USA) using the concentric cylinder geometry with double space at 25 °C at a constant speed of 62.1 s^−1^ (20 rpm). The relative viscosity (η*_rel_*) was calculated using Equation (16), the specific viscosity (η*_sp_*) with Equation (17), and the reduced viscosity (η*_red_*) with Equation (18):

(16) $$\eta_{rel}=\frac{\eta_{solution}}{\eta_{solvent}}$$

(17) $$\eta_{sp}=\eta_{rel}-1$$

(18) $$\eta_{red}=\frac{\eta_{sp}}{C}$$

where η*_solution_* is the viscosity of the pectin solution, η*_solvent_* is the viscosity of the solvent (100 mM NaCl solution), and *C* is the pectin concentration. The [η] was calculated by linear extrapolation of the reduced viscosity at zero concentration. The estimated [η] and the mean viscometric molecular weight (*MW_mean_*) were related through Equations (19) and (20), that is, the Mark–Houwink–Kuhn–Sakurada (MHKS) equation [12]:

(19) $$\left[\eta \right]=k{\left[MW_{mean}\right]}^{\propto }$$

(20) MWmean=([η]k)1∝

where the empirical constants *k* and *α* are usually taken from the literature with a similar temperature, solvent, and polymer used. However, values for Haden mango pectin were not found; therefore, the empirical constants’ values (K = 1.40 × 10^−6^ dL g^−1^; *α* = 1.34) were taken from a work where different mango varieties (Hoa Loc, Cat Chu, and Ghep) were used [37].

#### 2.6.8. Fourier-Transform Infrared Spectroscopy

For Fourier-transform infrared spectroscopy (FT-IR) analysis, a Nicolet 8700 FT-IR (Thermo Scientific, Waltham, MA, USA) equipment was used in a range of 4000–650 cm^−1^ wavelength, with 100 scans and a resolution of 4 cm^−1^. For analysis, 10 mg of sample were placed in a ZnSe crystal, and an absorbance mode with attenuated total reflection (ATR) and an optical speed of 0.4747 was used [38].

The esterification degree (%*DE*) of the pectin was also calculated using the diffuse reflectance infrared Fourier transform spectroscopy (DRIFTS) methodology to corroborate the results obtained by the titration method [39,40]. According to DRIFTS, the peak area values of free carboxyl groups (1630 cm^−1^) and the esterified groups (1745 cm^−1^) are related to the esterification degree by Equations (21) and (22):

(21) $$R=A_{1745}/\left(A_{1745}+A_{1630}\right)\times 100$$

(22) %DE=124.7×R+2.2013

#### 2.6.9. Thermal Analysis

For thermogravimetric analysis (TGA), a TGA 8000 thermogravimetric analyzer (Perkin Elmer, Waltham, MA, USA) with a derived thermogravimetry function (DTG) was used. In total, 10 mg of sample was placed in a platinum pan and heated from 40 °C to 600 °C at a rate of 10 °C/min, under a nitrogen atmosphere [41].

### 2.7. Power Consumption and Economic Analysis

The power and energy consumed, the specific energy consumption, and the cost of 1 kg of pectin was calculated as described elsewhere [42] with minor modifications. In brief, from the information noted in the equipment, it was possible to use Equation (23):

(23) $$E_{t}=\left(G\;\times \;t\right)/1000$$

where E_t_ stands for energy consumption (kJ), G for power consumption (W), and t for time (s).

From the information obtained by Equation (21), the cost per unit of electrical energy and the yield obtained in pectin extraction, it was possible to calculate the total energy consumption and the total expense energy to produce 1 kg of pectin considering the national average rate per kWh in Mexico. In addition, according to the yield of extraction, it was possible to estimate the amount and the cost of reactive to produce 1 kg of pectin. The summatory of the reactive and the energy cost was considered as the final cost of production. The cost was reported in USD assuming the equivalency of 1 USD = 20.31 MXN.

## 3. Results and Discussion

### 3.1. Pectin Extraction Yield

Throughout the transformation of Haden mango peel to flour, a yield of 95.65 ± 0.08% was achieved. To obtain a kinetic characterization of the pectin extraction, the yield of pectin from Haden mango peel flour was evaluated at the different temperatures and times proposed (Table 1). The obtained yields, ranging from 4.64 ± 0.62 to 13.82 ± 2.18%, were similar to pectin yields from other mango varieties, such as Tommy Atkins (10.05%), Alphonso (12.76%), and Honey (9.20%), obtained with HCl at 85 °C and hydrothermal microwave-assisted acid free extraction at 110 °C [43], and Kent mango (3.97–7.38%) extracted with ethanol and ammonium oxalate/oxalic acid at 85 °C [44]. Nevertheless, higher yields have been reported for Tainong mango pectin (16.70–17.15%) using citric acid and ultrasound-assisted extraction at 80 °C [18]. The variety of pectin yields could be explained not only by the mango cultivar, but also due to differences in temperatures, times, and type of acid in the extraction method, which, with increased energy, exposure time, and acid aggressiveness, could favor the degradation of pectin. In addition, some methods contained previous steps to facilitate the exposure of pectin and its extraction, such as enzymatic inactivation or ethanol washes [18,43,44]. 

Additionally, the extraction performed with citric acid as reported in this work could be considered friendlier to the environment and cheaper than other traditional extraction methods with sulfuric, hydrochloric, or nitric acid as it is a less aggressive acid and comes from citrus fruits, such as lemon, which makes it easy to obtain as well as biodegradable in the environment [12,19]. From the technological side, citric acid extraction of pectin has been reported to improve specific characteristics of the pectin, such as the molecular weight, viscosity, and methoxy groups, compared to pectin from the same source but extracted by different methods [20]. Organic acids like citric acid have a lower dissociation constant compared to mineral acids, so they have lower hydrolyzing capacity and depolymerization effect at the cost of extraction performance. Furthermore, the yield of pectin by acid extraction increases as the acid dissociation constant increases [20,45].

Therefore, the success of the extraction was evaluated not only by the yield obtained but also by the characteristics of the pectin obtained. 

### 3.2. Time and Temperature Effect on Extraction Yield

**Figure A1:**
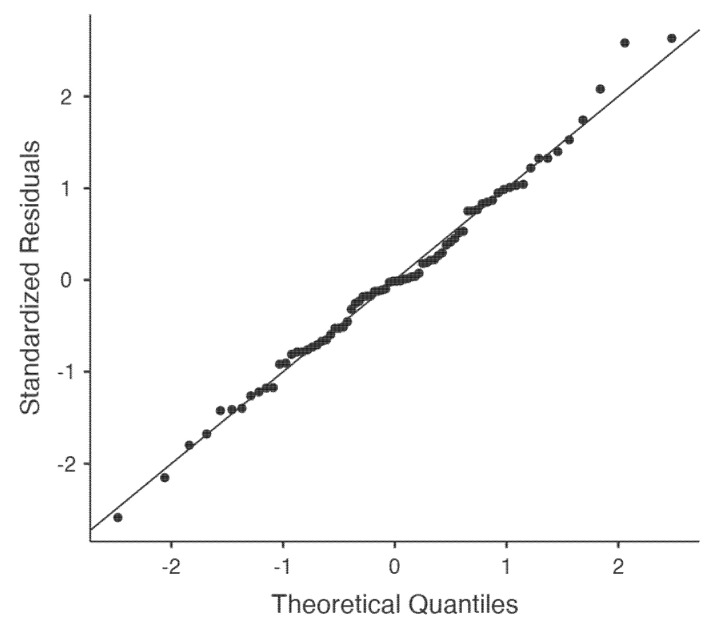
Q-Q plot for pectin yield extraction.

According to Table 2, it was observed that the yield increased with respect to the extraction time and temperature. Nevertheless, statistical analysis showed that temperature is the main factor that affected the pectin yield extraction (Appendix A Table A1, Table A2, Table A3, Table A4 and Table A5, Figure A1) since no significant differences between the time and temperature vs. time interaction were detected. This behavior may be explained by the fact that thermal degradation of pectin occurred, as a consequence of a prolonged time of exposure to high temperatures, causing de-polymerization to monosaccharides or oligosaccharides, which could be reflected in the low molecular weights [46,47]. It is also interesting to note that the yield increased as the temperature increased, possibly due to an increment in the solubility of the pectin before being degraded [25]. Results suggest that at higher temperatures, it is possible to extract more pectin from Haden mango peel flour, but at a certain time, despite the fact that there is an extraction of the pectin reflected in the extraction yield, the pectin also starts to be degraded, which generates a decrease in extraction performance, possibly because maximum solubility of the pectin has been achieved [25]. To prove this hypothesis, the kinetic and thermodynamic characterization were carried out from the mathematical model presented in Section 2.4 and Section 2.5.

### 3.3. Kinetic and Thermodynamic Characterization 

The parameters obtained after the kinetic characterization of the Haden mango peel pectin extraction are presented in Table 3. The results showed that the higher the temperature, the higher the total extractable pectin (*P_E_*), regardless of the time required to achieve it. Furthermore, the activation energy (*Ea*_1_) showed that increasing the temperature facilitated the pectin extraction process, while *Ea*_2_ indicated that a part of the pectin that was extracted began to degrade, despite the existence of an equilibrium semi-state in dissolved pectin [48]. Therefore, it was essential for the extraction process to stop the application of energy after a certain time (*t_max_*) and, with this, the degradation of pectin. Table 3 also shows that the activation energy for the degradation of pectin (−29.13 kJ/mol) was negative and lower than that required to dissolve it (5.53 kJ/mol), which indicates that it was not possible to extract pectin without degrading at least a part of it.

On the other hand, the thermodynamic parameters of the Haden mango peel pectin extraction process are indicated in Table 4, and support the data obtained for the Ea values of Table 3, as the dissolution process of pectin was endothermic (Δ*H*^#^_1_ = 2.64 kJ/mol) while its degradation was exothermic (Δ*H*^#^_2_ = −32.02 kJ/mol). The Δ*G*^#^ values are positives at all the temperatures, which indicates a temperature-dependent activation (Δ*G*^#^_1_) and a spontaneous thermal degradation in the pectin extracted (Δ*G*^#^_2_) that increases as the temperature rises. Both processes are irreversible according to the negative values of both Δ*S*^#^.

According to the data obtained at the evaluated temperatures, the process was spontaneous (Δ*G* < 0), dependent on temperature (Δ*H* > 0), and irreversible (Δ*S* > 0). 

**Figure 2:**
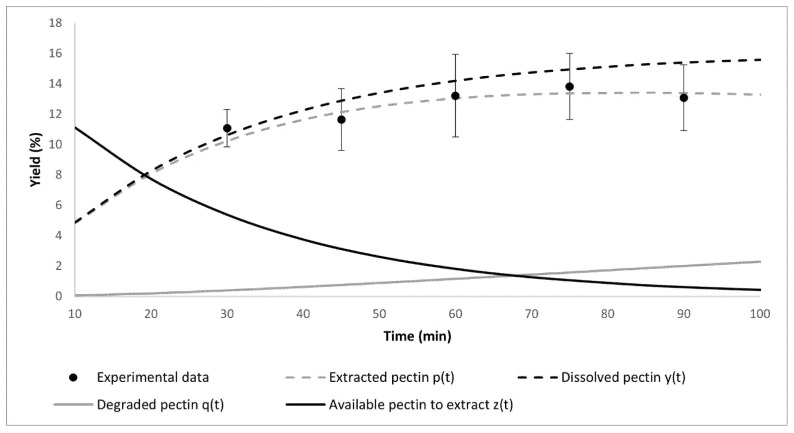
Changes in the different kinds of pectin according to time at 90 °C. *p*(*t*) stands for the extracted pectin, *y*(*t*) for the dissolved pectin, *q*(*t*) for degraded pectin, and *z*(*t*) for the available pectin to extract (also called protopectin). Experimental data are represented by black dots and error bars stands for standard error.

In addition, based on the extraction yield and its statistical analysis (Table 2), it was observed that the best extraction conditions evaluated were at 90 °C for 30 min (Figure 2), since longer extraction times did not show significant differences in yield. Furthermore, according to the kinetic and thermodynamic characterization, short times and higher temperatures were the best options for the pectin extraction. These results are similar to a previously reported pectin extraction using the same kinetic model [25], but from dragon fruit (*Hylocereus polurhizus*) at a temperature range of 30 to 80 °C and a time range of 30 to 240 min. Zaid et al. (2019) observed that higher-temperature extraction enhanced pectin yield but prolonged exposition caused degradation of itself. 

The pectin obtained from the extraction with the best evaluated conditions (90 °C for 30 min) was used for the chemical and physicochemical characterizations.

### 3.4. Physicochemical Characterization

#### 3.4.1. Chemical and Physicochemical Parameters 

Table 5 shows the results obtained from the chemical composition analysis, which serves as an indication of the general composition of the pectin obtained. The percentage of total carbohydrates found in Haden mango peel pectin (81.87 ± 0.44%) can be an indirect estimation of its conformation or purity (considering protein, ash, and moisture content), since pectin is a polysaccharide with various structural ramifications that contain disaccharides and protein residues (at lower presence, higher the pectin purity) that vary depending on the fruit species from which the pectin is obtained [49]. Furthermore, when compared with commercial pectin, it can be seen that the pectin obtained in this work had a higher moisture (11.76 ± 0.09%) and ash content (3.53 ± 0.02%) but lower protein content (0.83 ± 0.05%) than previously reported [50], which is indicative of pectin purity since the protein content has to be lower than 1.9% for commercial pectin [51]. The ash content could be associated with a higher mineral content (probably Na^+^, K^+^, Mg^2+^, Fe^2+^, Zn^2+^, or Ca^2+^) than the commercial one, which would affect the functional properties of pectin, such as gelling and cation-binding capacity [51,52]. Nevertheless, compared to other pectin sources (Table 4) like eggplant peel [32], hawthorn [50], black carrot pomace [42], or sour orange peel [53], Haden mango peel pectin was in the range of ash content and had a lower protein content, which is one indication of its purity. 

The percentages of esterification and methoxylation (Table 5) indicated that pectin can be classified as high methoxylation. Compared to other mango pectin, with DE in intervals between 85.43% and 88.38%, in those samples, a galacturonic acid content between 29.35% and 53.35% was reported [17,18], which is lower than the 71.57% found in the Haden mango peel pectin and is in the range accepted (>65%) by the food industry [50,54]. This also serves as an indicator of the pectin purity level for commercial applications [12,50]. The galacturonic acid content in the analyzed pectin could indicate that most of the structure is made up of the homogalacturonan region, i.e., that the Rhamnogalacturonan regions I and II appear in a smaller proportion in this pectin than the Homogalacturonan region. Additionally, methoxy groups ought to be found in the main chain in a great proportion due to its high %MeO (13.35; usually from 0.2 to 12), which is also an indicator of pectin’s ability to form gels and be combined with metallic ions [55].

On the other hand, the equivalent weight is an indicative parameter of quality and pectin degradation, which can be affected by the acid [56] and the heating time during pectin extraction [55]. The equivalent weight obtained for this pectin was 3657.55 ± 8.41 mg, which is higher than the values previously reported (1485.78 mg) for mango peel pectin from the Nam Dok Mai variety [57] and for other sources like *Dillenia indica* fruit (1025.32), cocoa husk (510.68 to 645.19 mg), passion fruit peel (781 to 826 mg), apple pomace (833.33 to 1666.30 mg) [56], and Malus domestica ‘Falticeni’ apple pomace (961 mg) [22]. The high equivalent weight obtained in this work is important because a higher equivalent weight is associated with a greater ability for pectin gel formation [56,57]. The equivalent weight is related to the amount of not esterified galacturonic acids in the molecular chains of pectin, which means that at a higher esterification degree, the equivalent weight will increase and the free acid content will decrease [58]. The obtained value for the equivalent weight could be explained not only by the high DE of the pectin but by the use of citric acid, which is less aggressive than the acids that are traditionally used for pectin extraction [36]. Another factor that could explain the high DE and equivalent weight of the pectin is the time of extraction, as long extraction times have been reported to cause depolymerization and deesterification of pectin [58]. Therefore, the short extraction time (30 min) used for pectin extraction could have caused a scarcity of breaks in the pectin main chain of polygalacturonic acid but a greater break on pectin side-chains [22,59]. A relatively weak acid extraction is also supported by the near to zero free acidity value (0.27 ± 0.00 mEq carboxyl free/g) found in the pectin, which is associated with the residual acid [12] and is usually inversely proportional to the equivalent weight. Furthermore, the obtained value is lower than the reported value for commercial pectin of 0.733 mEq carboxyl free/g [36]. 

According to the specific characteristics, that is, high DE (81.81%) and %MeO (13.35%), galacturonic acid content in the range of acceptance for industrial applications (71.57%), and high equivalent weight (3657.55 mg), Haden mango peel pectin could be used for different purposes, such as food, environmental, or pharmaceutical applications, where its gelling, emulsifier, and biodegradable characteristics could be appreciated [47,60]. For example, as a gelling agent [46], delivery systems [6], or as emulsifier [21]. In addition, higher DE can be associated with a better microcapsule formation ability in the meaning of thermal degradation stability and encapsulated retention [61].

#### 3.4.2. Monosaccharide Composition

According to 1-phenyl-3-methyl-5-pyrazolone (PMP) derivative analysis of saccharides, the following monosaccharides were found in Haden mango peel pectin: mannose, rhamnose, galacturonic acid, glucose, galactose, and arabinose. 

The results obtained in this work are in accordance with previously reported analyses of pectin by PMP derivatization in which the same monosaccharides were found [25,31]. Furthermore, a similar presence of sugar was reported by the HPLC-refractive index detector methodology [32,53]. However, in these analyses, pectin was obtained from other fruit sources, such as citrus, dragon fruit, or eggplant, and some also reported the presence of xylose and fructose. Both absences can be explained by the pectin source and by the scope of the PMP derivatization technique.

Although PMP derivatization provides sugars with strong absorbance under UV light, the technique has the following disadvantages: non-reducing and alcohol sugars, or some specific sugars, such as fructose, do not react with PMP due to the stability of its carbonyl group or by the lack of an aldehyde group. Furthermore, not all reducing sugars react in the same proportion to PMP (e.g., yields for mannose-PMP and galactose-PMP are higher than xylose-PMP yield) possibly due to their ring structures and the position of the covalent groups [62]. Therefore, the absence of some sugars, such as fructose, in PMP analysis does not necessarily mean that they are not present in Haden mango peel pectin. In the same sense, the percentage of every sugar will not accurately match the monosaccharide percentages obtained from other analyses due to yield differences in the PMP reaction. However, the results are valuable for estimation of the monosaccharide composition due to the qualitative detection of some sugars. Furthermore, PMP derivatization analysis can be coupled with other techniques, such as galacturonic acid content determination by titration or fructose content determination by reflectometry, for deeper characterization of the pectin composition as was done in this work. Therefore, the determination of the glucose + fructose content by reflectometry showed a concentration of 281 mg/L. The glucose assay showed a concentration of <1 mg/L, which allows an approximate estimation of the fructose concentration of 281 mg/L, which corresponds to 9.36% of the Haden mango peel pectin sample.

#### 3.4.3. Intrinsic Viscosity and Viscometric Molecular Weight

**Figure A2:**
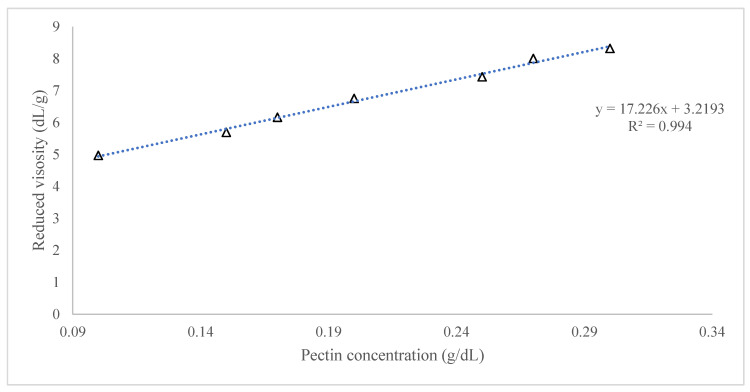
Reduced viscosity curve of Haden mango peel pectin.

The experimental viscosities obtained from mango pectin solutions in 0.1 M NaCl at different concentrations, as well as the relative, specific, and reduced viscosities calculated for each solution according to Section 2.6.7 are presented in Appendix A (Table A6, Figure A2).

An intrinsic viscosity of 3.2193 dL g^−1^ was obtained. With this value, and the constants for mango pectin used (K = 1.40 × 10^−6^ dL g^−1^; α = 1.34) [37], MW_mean_ = 55,909.76 g mol^−1^ or 55.91 kDa was calculated. The molecular weight obtained was within the previously reported average range for pectin (50–150 kDa), closer to the lower end of the range [11], which could be an indication of the degradation suffered by pectin during its extraction due to the temperature and its de-polymerization to monosaccharides or oligosaccharides [46,47]. The molecular weight is usually directly associated with the strength of the gel formed by the pectin, as the number of junction zones that can be formed per molecule affects the extent of cross-linking. However, low molecular weights do not affect pectin’s emulsifying properties or its ability to form hydrogels [16].

#### 3.4.4. FT-IR

**Figure 3:**
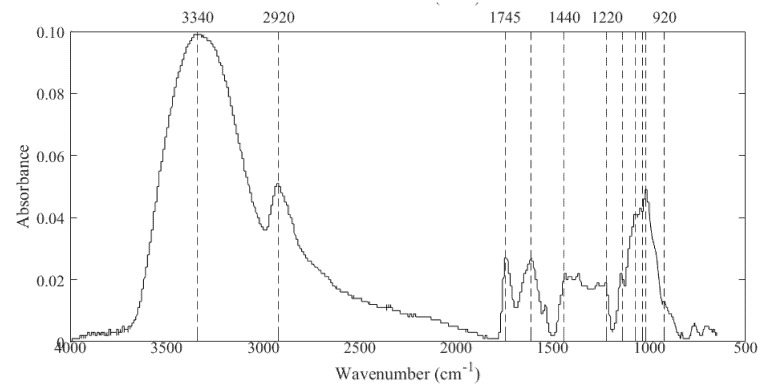
FT-IR spectrum of Haden mango peel pectin.

In the Fourier-transform infrared spectroscopy (FT-IR) spectrum of Haden mango peel pectin (Figure 3), it can be observed that the region of the functional groups (4000 to 1450 cm^−1^) corresponds to a typical spectrum of a polysaccharide. A broad and medium-depth signal was observed at around 3340 cm^−1^, which corresponds to O-H stretching vibration of inter and intramolecular hydrogen bonding of alcohol, carboxylic acids, and phenols groups commonly found in pectin, together with a signal at approximately 2920 cm^−1^ corresponding to the C-H stretching of alkane groups (CH_2_) [47,53,59]. 

FT-IR analysis can also be used to identify the characteristics of pectin bands in the fingerprint region. In this region, carboxyl group stretch signals were observed, centered on various wave numbers: C-O stretching in 1140, C-H stretching in 1070, C-O stretching in 1035, C-C stretching in 1018, and C-O bending in 920, and the representation of antisymmetric and symmetric stretching of ionic carboxyl groups from 1440 to 1220 cm^−1^ [57]. In general, the spectrum was similar to others reported for pectin from mangoes of different varieties (Amrapali, Fazlee, Langra, and Kharsapat), with the difference of a pronounced peak at 1018 cm^−1^, which in pectin from other mango varieties is less intense [63,64]. In addition, a peak centered at 1745 cm^−1^ was observed. This band is due to ester carbonyl groups’ (C=O) stretching vibration, which are characteristic of esterified pectin [57], and also seems to indicate the presence of hydrophobic groups, which allows the Haden mango peel pectin to be used as an emulsifying agent or in applications where hydrophobic capacity is required [47]. Furthermore, based on the area of 1745 cm^−1^ and 1630 cm^−1^ peaks (which corresponds to C=O stretching of the non-methylated carboxyl group) [47], it was found that Haden mango peel pectin can be classified as high esterification degree pectin (DE > 50%) as it was also found in the titration method [39,40]. 

#### 3.4.5. Thermal Analysis

**Figure 4:**
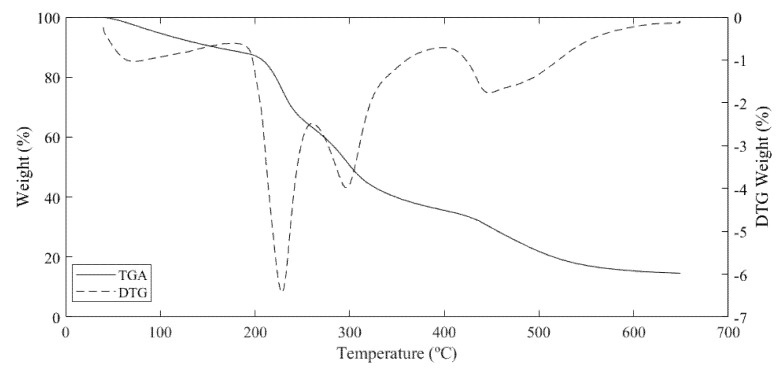
TGA and DTG curves of Haden mango peel pectin.

In the thermogravimetric analysis (TGA) and derived thermogravimetry (DTG) curves of Haden mango peel pectin (Figure 4), a main weight loss was observed with a peak centered at 230 °C after loss of free water (100 to 200 °C), which could be attributed to dehydration of the carbohydrate units followed by pyrolytic depolymerization and decomposition of the pectin molecules as temperature increases [47,65]. Furthermore, another peak centered at 300 °C can be observed, which could be due to impurities (possibly proteins), starch residues, or pectin-bound oligosaccharides [43]. 

Finally, in accordance with TGA curves, a loss of approximately 15% of weight was displayed from 50 to 210 °C, which corresponds to water evaporation equivalent to the moisture content determined in the chemical analysis. A weight loss of approximately 35% was observed from 210 to 300 °C, possibly attributed to pectin structure degradation [42]. Another loss of weight (approximately 15%) could be observed from 300 to 420 °C possibly due to residual low-molecular polysaccharides bound to the pectin and to oxidative degradation of pectin [43,65]. 

### 3.5. Power Consumption and Economic Analysis

The final cost per kg of Haden mango peel pectin, according to Table 6 and Table 7, was calculated on 1045.79 MXN or 51.49 USD. The commercial citrus pectin cost varies from 32 USD (for domestic use) to 289 USD (reactive degree) per kg approximately, which makes it possible to find a niche in the market for Haden mango peel pectin.

The pectin global market had a compound annual growth rate of 5.7% from 2014 to 2019 [66]. According to the tendency, an increase in the market size value from 1 billion USD in 2019 to 1.5 billion USD by 2025 was expected [67]. However, due to coronavirus disease (COVID-19) and the growing popularity of natural and organic products/ingredients, the demand for pectin raised the manufacture of functional and nutritious food products and it is expected to continue growing in the next years [66].

Therefore, exploration of the pectin global market is very attractive, with new sources of pectin. However, in the case of Haden mango peel pectin, the results presented in this section were calculated at the laboratory scale and only based on energy consumption and reactive requirements, i.e., there is still a wide range of improvement for a scale-up of the process, with further optimization and pilot-scale works needed. 

## 4. Conclusions

In this work, it was possible to establish a quick (30 min) pectin mild acid extraction from Haden mango peel flour using citric acid at 90 °C, obtaining an average yield of 11.08 ± 1.23%. The physicochemical characteristics of the pectin show it is suitable for potential use in food, pharmaceutical, or environmental applications, although further purification of the pectin could be done.

In addition, the kinetic and thermodynamic information of the pectin extraction process may be useful for scaling and optimization purposes, not only as a scaling criterion, but also to compare the performance of the process in terms of equilibrium conversions and system limitations.

The activation energy required to dissolve the pectin (5.53 kJ/mol) and to degrade it (−29.13 kJ/mol) indicates that after a time *t_max_*, the application of energy must be stopped, to interrupt the degradation reaction and proceed to pectin recovery. This data is supported by the physicochemical characteristics of the pectin, indicating degradation of the main chains even with the use of relatively mild acid extraction. Therefore, it can be concluded that during the extraction of Haden mango pectin through the application of energy, there will always be a fraction of pectin in degradation, which supports a fast extraction at high temperatures to obtain the greatest amount of extractable pectin with the least possible thermal damage. 

Finally, the economic analysis at the laboratory scale indicates that it is worth exploring the optimization and scale-up of the process in future works. 

## Figures and Tables

**Table 1:** Mobile phase gradients for UPLC analysis.

Time (min)	A (%)	B (%)
0	100	0
5	80	20
27	55	45
30	90	10

**Table 2:** Haden mango peel pectin extraction yields at the different evaluated conditions.

Temperature (°C)	Time (min)	Yield (%)
60 ^(a)^	30	4.64 ± 0.62
	45	6.19 ± 0.84
	60	6.80 ± 0.40
	75	7.03 ± 0.77
	90	6.38 ± 0.21
70 ^(ab)^	30	6.90 ± 0.73
	45	7.76 ± 0.47
	60	8.61 ± 1.09
	75	8.57 ± 0.93
	90	7.61 ± 0.80
80 ^(b)^	30	7.37 ± 1.82
	45	7.93 ± 2.19
	60	9.41 ± 0.62
	75	10.62 ± 1.62
	90	10.02 ± 1.08
90 ^(c)^	30	11.08 ± 1.23
	45	11.65 ± 2.04
	60	13.22 ± 2.73
	75	13.82 ± 2.18
	90	13.07 ± 2.17

Note: Different letters indicate significant differences between temperature treatments, *p* < 0.05.

**Table 3:** Kinetic parameters obtained at different temperatures during Haden mango peel pectin extraction.

Temperature (°C)	*P_E_* (%)	*k*_1_ (min^−1^)	*k*_2_ (min^−1^)	*t_max_* (min)	*Y_max_* (%)	R^2^	*Ea*_1_ (kJ/mol)	*Ea*_2_ (kJ/mol)
60	9.00	0.02958	0.00349	81.89	7.93	0.8778	5.53	−29.13
70	11.00	0.03333	0.00397	72.45	9.69	0.8234		
80	11.66	0.03118	0.00100	113.97	10.79	0.8688		
90	16.00	0.03635	0.00213	82.89	14.39	0.9213		

Note: *Pe* = Total extractable pectin; *k* = Velocity constant; *t_max_ =* Extraction time for maximum yield; *Y_max_* = Maximum theorical yield; *Ea* = Activation energy; and R^2^ = Correlation coefficient.

**Table 4:** Thermodynamic parameters obtained at different temperatures during Haden mango peel pectin extraction.

Temperature (°C)	Δ*H*^#^_1_ (kJ/mol)	Δ*H*^#^_2_ (kJ/mol)	Δ*S*^#^_1_ (kJ/mol)	Δ*S*^#^_2_ (kJ/mol)	Δ*G*^#^_1_ (kJ/mol)	Δ*G*^#^_2_ (kJ/mol)	*F*	Δ*H* (kJ/mol)	Δ*S* (kJ/mol)	Δ*G* (kJ/mol)
60	2.64	−32.02	−0.07	−0.19	25.83	31.67	8.47	34.66	0.12	−5.84
70					26.52	33.58	8.39			−7.06
80					27.22	35.49	31.18			−8.28
90					27.92	37.41	17.05			−9.49

Note: Δ*H*^#^ = Activation enthalpy; Δ*S*^#^ = Activation entropy; Δ*G*^#^ = Gibbs free energy of activation; *F* = Equilibrium constant; Δ*H* = Enthalpy; Δ*S* = Entropy; and Δ*G* = Gibbs free energy.

**Table 5:** Chemical and physicochemical parameters of Haden mango peel pectin compared to pectin from different sources.

Variable		Pectin Source						
	Haden Mango (*Mangifera indica* L.) Peel	Commercial	Commercial	Hawthorn (*Crataegus* spp.)	Eggplant (*Solanum melongena*) Peel	Black Carrot (*Daucus carota* L.) Pomace	Sour Orange (*Citrus aurantium* L.) Peel	Nam Dok Mai Mango (*Mangifera indica* L.) Peel
Moisture (%)	11.76 ± 0.09	10.49 ± 0.04	7.10 ± 1.90	4.50 ± 0.80	5.85 ± 0.27	5.90 ± 1.30	8.81 ± 0.68	NR
Ash (%)	3.53 ± 0.02	NR	0.80 ± 0.10	0.20 ± 0.00	9.03 ± 1.09	2.80 ± 0.40	1.89 ± 0.51	NR
Protein (%)	0.83 ± 0.05	NR	2.50 ± 0.10	3.50 ± 0.10	9.13 ± 0.17	NR	1.45 ± 0.23	NR
Degree of esterification (%)	81.81 ± 0.00	82.29 ± 0.16	72.5 ± 0.20	78.20 ± 0.60	68.18 ±1.19	45.20 ± 5.00	6.77 ± 0.43	77.19 ± 0.72
Methoxyl percentage (%)	13.35 ± 0.00	13.42 *	11.8 ± 0.00	12.70 ± 0.10	11.13 *	7.38 *	NR	12.59 *
Galacturonic acid (%)	71.57 ± 1.26	NR	71.2 ± 1.30	86.70 ± 6.90	67.40	DU	65.30	NR
Free acidity (mEq carboxyl free/g)	0.27 ± 0.00	0.733 ± 0.00	NR	NR	NR	NR	NR	NR
Equivalent weight (mg)	3657.55 ± 8.41	1364.63 ± 0.00	NR	NR	NR	NR	NR	1485.78 ± 0.74
Reference	Present work	Ayora-Talavera et al., 2017	Cuevas-Bernardino et al., 2016		Kazemi et al., 2019	Sucheta et al., 2020	Hosseini et al., 2019	Wongkaew et al., 2020

Note: NR stands for not reported. DU stands for reported with different units. * Calculated according to the methodology used in this work using the reported DE data, for comparison purposes.

**Table 6:** Power consumption and economic analysis to produce 1 kg of Haden mango peel pectin.

Parameter		Value
Yield (%)		11.08
Power consumption (W)	Tray dehydrator	120
	Hammer grill	2800
	Heating plate	120
	Centrifuge	1400
	Refrigerator	150
	Vacuum oven	1400
	Coffee mill	110
Time (s)	Tray dehydrator	28,800
	Hammer grill	3600
	Heating plate	1800
	Centrifuge	900
	Refrigerator	43,200
	Vacuum oven	28,800
	Coffee mill	600
Energy (kJ)	Tray dehydrator	3456
	Hammer grill	10,080
	Heating plate	216
	Centrifuge	1260
	Refrigerator	6480
	Vacuum oven	40,320
	Coffee mill	66
Specific Energy (kJ/kg)		558,465.70
Price per unit of electricity (MXN/kWh)		2.58
Cost of energy per unit of pectin (MXN/kg)		400.23
Cost of energy per unit of pectin (USD/kg)		19.71

**Table 7:** Reactive requirements and economic analysis to produce 1 kg of Haden mango peel pectin.

Parameter	Reactive		
	Citric Acid	Ethanol	Purified Water
Cost (MXN/kg or L)	60	45	0.5
Required quantity to produce 1 kg of Haden mango peel pectin (kg or L)	1.73	9.03	270.76
Cost/kg of pectin (MXN)	104.04	406.14	135.38
Cost/kg of pectin (USD)	5.12	20.00	6.67

## Data Availability

Data is contained within the article.

## Appendix A

**Table A1:** ANOVA for pectin yield extraction.

ANOVA					
	Sum of Squares	df	Mean Square	F	*p*
Time	54.49	4	13.623	2.139	0.088
Temperature	362.18	3	120.727	18.957	<0.001
Time*Temperature	9.77	12	0.814	0.128	1
Residuals	356.64	56	6.369		

**Table A2:** Tukey test for temperature treatments in pectin yield extraction.

Post Hoc Comparisons—Temperature							
Comparison							
Temperature		Temperature	Mean Difference	SE	df	t	p_tukey_
60	-	70	−1.68	0.787	56	−2.13	0.155
	-	80	−2.86	0.842	56	−3.4	0.007
	-	90	−6.36	0.867	56	−7.34	<0.001
70	-	80	−1.18	0.822	56	−1.44	0.482
	-	90	−4.68	0.847	56	−5.53	<0.001
80	-	90	−3.5	0.898	56	−3.9	0.001

**Table A3:** Tukey test for time treatments in pectin yield extraction.

Post Hoc Comparisons—Time							
Comparison							
Time		Time	Mean Difference	SE	df	t	p_tukey_
30	-	45	−0.881	0.929	56	−0.948	0.877
	-	60	−2.009	0.953	56	−2.108	0.231
	-	75	−2.51	0.977	56	−2.568	0.09
	-	90	−1.77	0.946	56	−1.87	0.345
45	-	60	−1.129	0.918	56	−1.229	0.734
	-	75	−1.629	0.943	56	−1.728	0.426
	-	90	−0.889	0.911	56	−0.977	0.865
60	-	75	−0.5	0.967	56	−0.517	0.985
	-	90	0.239	0.936	56	0.256	0.999
75	-	90	0.74	0.96	56	0.77	0.938

**Table A4:** Tukey test for time*temperature treatments in pectin yield extraction.

Post Hoc Comparisons—Time*Temperature									
Comparison									
Time	Temperature		Time	Temperature	Mean Difference	SE	df	t	p_tukey_
30	60	-	30	70	−2.2583	1.93	56	−1.1717	1
		-	30	80	−2.7259	1.78	56	−1.5276	0.991
		-	30	90	−6.439	1.93	56	−3.3407	0.129
		-	45	60	−1.5414	1.78	56	−0.8638	1
		-	45	70	−3.1124	1.63	56	−1.9106	0.922
		-	45	80	−3.2832	1.93	56	−1.7034	0.972
		-	45	90	−7.0087	1.78	56	−3.9277	0.028
		-	60	60	−2.1547	1.78	56	−1.2075	0.999
		-	60	70	−3.9649	1.69	56	−2.3421	0.698
		-	60	80	−4.7648	1.93	56	−2.4721	0.608
		-	60	90	−8.5755	1.93	56	−4.4492	0.006
		-	75	60	−2.3884	1.69	56	−1.4108	0.996
		-	75	70	−3.9249	1.69	56	−2.3185	0.714
		-	75	80	−5.9718	1.93	56	−3.0983	0.22
		-	75	90	−9.1766	2.19	56	−4.1988	0.013
		-	90	60	−1.7338	1.93	56	−0.8996	1
		-	90	70	−2.962	1.78	56	−1.6599	0.978
		-	90	80	−5.3775	1.78	56	−3.0135	0.261
		-	90	90	−8.4299	1.78	56	−4.7241	0.002
	70	-	30	80	−0.4676	1.93	56	−0.2426	1
		-	30	90	−4.1807	2.06	56	−2.029	0.876
		-	45	60	0.7169	1.93	56	0.3719	1
		-	45	70	−0.8541	1.78	56	−0.4786	1
		-	45	80	−1.0249	2.06	56	−0.4974	1
		-	45	90	−4.7504	1.93	56	−2.4646	0.614
		-	60	60	0.1037	1.93	56	0.0538	1
		-	60	70	−1.7066	1.84	56	−0.926	1
		-	60	80	−2.5065	2.06	56	−1.2164	0.999
		-	60	90	−6.3172	2.06	56	−3.0659	0.235
		-	75	60	−0.1301	1.84	56	−0.0706	1
		-	75	70	−1.6666	1.84	56	−0.9043	1
		-	75	80	−3.7135	2.06	56	−1.8022	0.952
		-	75	90	−6.9183	2.3	56	−3.0031	0.266
		-	90	60	0.5245	2.06	56	0.2545	1
		-	90	70	−0.7036	1.93	56	−0.3651	1
		-	90	80	−3.1192	1.93	56	−1.6183	0.983
		-	90	90	−6.1716	1.93	56	−3.202	0.177
	80	-	30	90	−3.7132	1.93	56	−1.9265	0.917
		-	45	60	1.1844	1.78	56	0.6638	1
		-	45	70	−0.3865	1.63	56	−0.2373	1
		-	45	80	−0.5574	1.93	56	−0.2892	1
		-	45	90	−4.2829	1.78	56	−2.4001	0.659
		-	60	60	0.5712	1.78	56	0.3201	1
		-	60	70	−1.2391	1.69	56	−0.7319	1
		-	60	80	−2.0389	1.93	56	−1.0578	1
		-	60	90	−5.8497	1.93	56	−3.035	0.25
		-	75	60	0.3375	1.69	56	0.1994	1
		-	75	70	−1.199	1.69	56	−0.7083	1
		-	75	80	−3.246	1.93	56	−1.6841	0.975
		-	75	90	−6.4507	2.19	56	−2.9516	0.293
		-	90	60	0.992	1.93	56	0.5147	1
		-	90	70	−0.2361	1.78	56	−0.1323	1
		-	90	80	−2.6516	1.78	56	−1.486	0.993
		-	90	90	−5.704	1.78	56	−3.1965	0.179
	90	-	45	60	4.8976	1.93	56	2.541	0.56
		-	45	70	3.3266	1.78	56	1.8642	0.936
		-	45	80	3.1558	2.06	56	1.5316	0.99
		-	45	90	−0.5697	1.93	56	−0.2956	1
		-	60	60	4.2844	1.93	56	2.2228	0.775
		-	60	70	2.4741	1.84	56	1.3425	0.998
		-	60	80	1.6742	2.06	56	0.8125	1
		-	60	90	−2.1365	2.06	56	−1.0369	1
		-	75	60	4.0507	1.84	56	2.1979	0.789
		-	75	70	2.5142	1.84	56	1.3642	0.998
		-	75	80	0.4672	2.06	56	0.2267	1
		-	75	90	−2.7375	2.3	56	−1.1883	1
		-	90	60	4.7052	2.06	56	2.2835	0.737
		-	90	70	3.4771	1.93	56	1.804	0.952
		-	90	80	1.0615	1.93	56	0.5507	1
		-	90	90	−1.9908	1.93	56	−1.0329	1
45	60	-	45	70	−1.571	1.63	56	−0.9644	1
		-	45	80	−1.7418	1.93	56	−0.9037	1
		-	45	90	−5.4673	1.78	56	−3.0639	0.236
		-	60	60	−0.6132	1.78	56	−0.3437	1
		-	60	70	−2.4235	1.69	56	−1.4316	0.996
		-	60	80	−3.2234	1.93	56	−1.6724	0.976
		-	60	90	−7.0341	1.93	56	−3.6495	0.06
		-	75	60	−0.8469	1.69	56	−0.5003	1
		-	75	70	−2.3834	1.69	56	−1.4079	0.996
		-	75	80	−4.4304	1.93	56	−2.2986	0.727
		-	75	90	−7.6351	2.19	56	−3.4935	0.089
		-	90	60	−0.1924	1.93	56	−0.0998	1
		-	90	70	−1.4205	1.78	56	−0.7961	1
		-	90	80	−3.8361	1.78	56	−2.1497	0.817
		-	90	90	−6.8884	1.78	56	−3.8603	0.034
	70	-	45	80	−0.1708	1.78	56	−0.0957	1
		-	45	90	−3.8963	1.63	56	−2.3919	0.665
		-	60	60	0.9577	1.63	56	0.5879	1
		-	60	70	−0.8525	1.53	56	−0.5579	1
		-	60	80	−1.6524	1.78	56	−0.926	1
		-	60	90	−5.4631	1.78	56	−3.0615	0.237
		-	75	60	0.724	1.53	56	0.4738	1
		-	75	70	−0.8125	1.53	56	−0.5317	1
		-	75	80	−2.8594	1.78	56	−1.6024	0.985
		-	75	90	−6.0642	2.06	56	−2.943	0.298
		-	90	60	1.3786	1.78	56	0.7725	1
		-	90	70	0.1504	1.63	56	0.0924	1
		-	90	80	−2.2651	1.63	56	−1.3905	0.997
		-	90	90	−5.3175	1.63	56	−3.2643	0.154
	80	-	45	90	−3.7255	1.93	56	−1.9329	0.914
		-	60	60	1.1286	1.93	56	0.5855	1
		-	60	70	−0.6817	1.84	56	−0.3699	1
		-	60	80	−1.4816	2.06	56	−0.719	1
		-	60	90	−5.2923	2.06	56	−2.5684	0.54
		-	75	60	0.8949	1.84	56	0.4855	1
		-	75	70	−0.6416	1.84	56	−0.3482	1
		-	75	80	−2.6886	2.06	56	−1.3048	0.999
		-	75	90	−5.8933	2.3	56	−2.5582	0.547
		-	90	60	1.5494	2.06	56	0.752	1
		-	90	70	0.3213	1.93	56	0.1667	1
		-	90	80	−2.0943	1.93	56	−1.0866	1
		-	90	90	−5.1466	1.93	56	−2.6702	0.469
	90	-	60	60	4.8541	1.78	56	2.7202	0.435
		-	60	70	3.0438	1.69	56	1.798	0.953
		-	60	80	2.2439	1.93	56	1.1642	1
		-	60	90	−1.5668	1.93	56	−0.8129	1
		-	75	60	4.6204	1.69	56	2.7293	0.429
		-	75	70	3.0839	1.69	56	1.8217	0.948
		-	75	80	1.0369	1.93	56	0.538	1
		-	75	90	−2.1678	2.19	56	−0.9919	1
		-	90	60	5.2749	1.93	56	2.7368	0.424
		-	90	70	4.0468	1.78	56	2.2678	0.747
		-	90	80	1.6312	1.78	56	0.9141	1
		-	90	90	−1.4211	1.78	56	−0.7964	1
60	60	-	60	70	−1.8103	1.69	56	−1.0693	1
		-	60	80	−2.6101	1.93	56	−1.3542	0.998
		-	60	90	−6.4209	1.93	56	−3.3313	0.132
		-	75	60	−0.2337	1.69	56	−0.1381	1
		-	75	70	−1.7702	1.69	56	−1.0457	1
		-	75	80	−3.8172	1.93	56	−1.9805	0.897
		-	75	90	−7.0219	2.19	56	−3.213	0.173
		-	90	60	0.4208	1.93	56	0.2183	1
		-	90	70	−0.8073	1.78	56	−0.4524	1
		-	90	80	−3.2229	1.78	56	−1.8061	0.951
		-	90	90	−6.2752	1.78	56	−3.5166	0.084
	70	-	60	80	−0.7999	1.84	56	−0.434	1
		-	60	90	−4.6106	1.84	56	−2.5017	0.587
		-	75	60	1.5765	1.6	56	0.9878	1
		-	75	70	0.0401	1.6	56	0.0251	1
		-	75	80	−2.0069	1.84	56	−1.089	1
		-	75	90	−5.2117	2.11	56	−2.4683	0.611
		-	90	60	2.2311	1.84	56	1.2106	0.999
		-	90	70	1.003	1.69	56	0.5925	1
		-	90	80	−1.4126	1.69	56	−0.8344	1
		-	90	90	−4.4649	1.69	56	−2.6375	0.492
	80	-	60	90	−3.8107	2.06	56	−1.8494	0.94
		-	75	60	2.3764	1.84	56	1.2895	0.999
		-	75	70	0.8399	1.84	56	0.4557	1
		-	75	80	−1.207	2.06	56	−0.5858	1
		-	75	90	−4.4118	2.3	56	−1.9151	0.92
		-	90	60	3.031	2.06	56	1.471	0.994
		-	90	70	1.8028	1.93	56	0.9354	1
		-	90	80	−0.6127	1.93	56	−0.3179	1
		-	90	90	−3.6651	1.93	56	−1.9015	0.925
	90	-	75	60	6.1872	1.84	56	3.3572	0.124
		-	75	70	4.6507	1.84	56	2.5235	0.572
		-	75	80	2.6037	2.06	56	1.2636	0.999
		-	75	90	−0.601	2.3	56	−0.2609	1
		-	90	60	6.8417	2.06	56	3.3204	0.135
		-	90	70	5.6136	1.93	56	2.9125	0.315
		-	90	80	3.198	1.93	56	1.6592	0.978
		-	90	90	0.1457	1.93	56	0.0756	1
75	60	-	75	70	−1.5365	1.6	56	−0.9627	1
		-	75	80	−3.5835	1.84	56	−1.9444	0.91
		-	75	90	−6.7882	2.11	56	−3.215	0.172
		-	90	60	0.6545	1.84	56	0.3552	1
		-	90	70	−0.5736	1.69	56	−0.3388	1
		-	90	80	−2.9891	1.69	56	−1.7657	0.96
		-	90	90	−6.0415	1.69	56	−3.5688	0.074
	70	-	75	80	−2.047	1.84	56	−1.1107	1
		-	75	90	−5.2517	2.11	56	−2.4873	0.598
		-	90	60	2.191	1.84	56	1.1889	1
		-	90	70	0.9629	1.69	56	0.5688	1
		-	90	80	−1.4527	1.69	56	−0.8581	1
		-	90	90	−4.505	1.69	56	−2.6611	0.475
	80	-	75	90	−3.2047	2.3	56	−1.3911	0.997
		-	90	60	4.238	2.06	56	2.0568	0.864
		-	90	70	3.0099	1.93	56	1.5616	0.988
		-	90	80	0.5943	1.93	56	0.3083	1
		-	90	90	−2.458	1.93	56	−1.2753	0.999
	90	-	90	60	7.4427	2.3	56	3.2308	0.166
		-	90	70	6.2146	2.19	56	2.8436	0.356
		-	90	80	3.7991	2.19	56	1.7383	0.966
		-	90	90	0.7467	2.19	56	0.3417	1
90	60	-	90	70	−1.2281	1.93	56	−0.6372	1
		-	90	80	−3.6437	1.93	56	−1.8904	0.928
		-	90	90	−6.696	1.93	56	−3.4741	0.094
	70	-	90	80	−2.4156	1.78	56	−1.3537	0.998
		-	90	90	−5.4679	1.78	56	−3.0642	0.236
	80	-	90	90	−3.0524	1.78	56	−1.7105	0.971

**Table A5:** Shapiro–Wilk normality test for pectin yield extraction.

Normality Test (Shapiro-Wilk)	
Statistic	*p*
0.991	0.88

Table A6 shows the experimental viscosities obtained from mango pectin solutions in 0.1 M NaCl at different concentrations, as well as the relative, specific, and reduced viscosities calculated for each solution.

**Table A6:** Experimental and calculated viscosities of Haden mango peel pectin.

Solution	Viscosity (Pa.s)	Relative Viscosity	Specific Viscosity	Reduced Viscosity
Solvente NaCl (0.1 M)	8.05750 × 10^−4^	-	-	-
0.1%	1.20634 × 10^−3^	1.49717	4.97167 × 10^−1^	4.971668981
0.15%	1.49367 × 10^−3^	1.85376	8.53758 × 10^−1^	5.691721544
0.17%	1.65053 × 10^−3^	2.048441847	1.04844	6.167304982
0.20%	1.89412 × 10^−3^	2.35076	1.35076	6.753783007
0.25%	2.30238 × 10^−3^	2.857433846	1.85743	7.429735385
0.27%	2.54787 × 10^−3^	3.162115961	2.16212	8.007836894
0.30%	2.81661 × 10^−3^	3.49564	2.49564	8.318812952

Figure A2 was plotted with the reduced viscosity data and, therefore, the equation of the line could be obtained, which when extrapolated to 0 allows an intrinsic viscosity equal to 3.2193 dL g^−1^ to be obtained. With the intrinsic viscosity and the constants for pectin used (K = 1.40 × 10^−6^ dL g^−1^; α = 1.34), an average molecular weight equal to 55.91 kDa was obtained.
